# Graft Materials and Biologics for Spinal Interbody Fusion

**DOI:** 10.3390/biomedicines7040075

**Published:** 2019-09-26

**Authors:** Marissa D’Souza, Nicholas A. Macdonald, Julian L. Gendreau, Pate J. Duddleston, Austin Y. Feng, Allen L. Ho

**Affiliations:** 1 School of Medicine, Mercer University School of Medicine, Macon, GA 31207, USA; marissa.dsouza@live.mercer.edu (M.D.); Nicholas.Aubrey.Macdonald@live.mercer.edu (N.A.M.); Julian.Lassiter.Gendreau@live.mercer.edu (J.L.G.); Pate.Jackson.Duddleston@live.mercer.edu (P.J.D.); 2 Department of Neurosurgery, Stanford University School of Medicine, Stanford, CA 94305, USA; fenga@stanford.edu

**Keywords:** allograft, autograft, biologics, bone-morphogenetic protein, ceramic, demineralized bone matrix, spinal fusion, genetic therapy, graft, mesenchymal stem cells

## Abstract

Spinal fusion is the most widely performed procedure in spine surgery. It is the preferred treatment for a wide variety of pathologies including degenerative disc disease, spondylolisthesis, segmental instability, and deformity. Surgeons have the choice of fusing vertebrae by utilizing cages containing autografts, allografts, demineralized bone matrices (DBMs), or graft substitutes such as ceramic scaffolds. Autografts from the iliac spine are the most commonly used as they offer osteogenic, osteoinductive, and osteoconductive capabilities, all while avoiding immune system rejection. Allografts obtained from cadavers and living donors can also be advantageous as they lack the need for graft extraction from the patient. DBMs are acid-extracted organic allografts with osteoinductive properties. Ceramic grafts containing hydroxyapatite can be readily manufactured and are able to provide osteoinductive support while having a long shelf life. Further, bone-morphogenetic proteins (BMPs), mesenchymal stem cells (MSCs), synthetic peptides, and autologous growth factors are currently being optimized to assist in improving vertebral fusion. Genetic therapies utilizing viral transduction are also currently being devised. This review provides an overview of the advantages, disadvantages, and future directions of currently available graft materials. The current literature on growth factors, stem cells, and genetic therapy is also discussed.

## 1. Introduction

Interbody fusion is an established treatment option for a wide range of spinal pathologies including degenerative disc disease, herniated discs, spondylolisthesis, infections, deformity, and neoplasia with the primary goal of providing spinal stabilization [1]. With over 400,000 cases performed in the United States each year, interbody fusion is the most commonly performed spinal surgery [2]. Fusions are performed on the cervical, thoracic, and lumbar spine. There are numerous methods by which fusions can be performed. These approaches include anterior and posterior approaches to the cervical spine, transpedicular, costotransversectomy, lateral extracavitary, and intrathoracic approaches to the thoracic spine, anterior (ALIF), posterior (PLIF), transforaminal (TLIF), and lateral (XLIF, DLIF, OLIF) interbody approaches to the lumbar spine. There is currently no definitive evidence advocating one approach spinal fusion over the other, and the choice for the appropriate approach is largely dictated by the specific pathologies being treated.

Spinal fusion surgeries involve the placement of an interbody device in a disc space or corpectomy cavity such as a cage, spacer, or structural graft to promote bony fusion [3]. For grafting, there are several different types of grafts that can be chosen for placement. These options include autografts, allografts, demineralized bone matrices (DBM) and/or graft substitutes such as ceramic scaffolding products. In addition, various mesenchymal stem cells (MSCs), growth factors, and synthetic peptides are being utilized to optimize fusion rates. Genetic therapies utilizing the capabilities of viral transduction are also currently being devised [4]. This review provides a succinct overview of each bone graft material including their advantages, disadvantages, and future directions for innovation. We also discuss the current literature of growth factors, stem cells, and genetic therapy [4,5,6,7,8].

## 2. Physiology of Bone Growth and Remodeling

With a wide variety of biologics on the market, it is important to understand not only the nature of bone grafts but also the properties of bone healing in order to select the most appropriate option for each patient undergoing surgery. Previous researchers have defined the three pillars of bone regeneration to be osteogenesis, osteoinduction, and osteoconduction. Osteogenesis is the synthesis of new bone cells derived from either the graft or the host. Osteoinduction is the process by which MSCs are recruited to differentiate into chondroblasts and osteoblasts. These newly formed osteoblasts are responsible for bone formation [9]. Finally, osteoconduction is the process by which capillaries, perivascular tissue, and MSCs form a scaffold along the graft, ultimately resulting in the fusion of the graft with host’s local bone [10]. The functionality of bone grafts in promoting spinal fusion is largely dependent on the graft’s ability to perform these three processes.

Not only do these processes need to occur, but a set timeline must be followed in order for the fusion to be successful. The three distinct stages of this timeline include the inflammatory stage, the repair stage, and the late remodeling stage. The inflammatory stage (hours to days, post-operatively) is predominated by the recruitment of inflammatory cells, osteoprogenitor cells, and fibroblasts via prostaglandins. The repair stage (weeks to months, post-operatively) is predominated by the formation of vascular ingrowths and collagen matrices via fibroblasts. Finally, the late-remodeling stage (months to years, post-operatively) is predominated by the restoration of bone to its baseline strength via mechanical stress over time. All three of these stages are critical for achieving successful fusion [11].

## 3. Bone Grafts

### 3.1. Autogenous Bone Grafts

Autogenous bone graft is currently the ‘gold standard’ for spinal fusion grafting and involves the transplantation of host bone from one anatomical site on a patient to another site. While harvesting sites can include the proximal tibia, distal femur, fibula, ribs, distal radius, and local bone harvested from spinal elements during surgery, the most common site to be utilized outside of the immediate surgical bed is the iliac crest [12]. Because the bone is extracted from the same individual patient, there is complete histocompatibility and no opportunity for infection from a graft donor. Autografts also have the advantage of having all three of the pillars of bone regeneration, including osteogenic, osteoinductive, and osteoconductive capabilities that other grafts may or may not offer.

Autografts can be further classified by bone type: cortical and cancellous. Cortical bone is described as an extremely dense bone with limited porosity, while cancellous bone is the opposite and is extremely porous [10]. While cortical bone provides the advantage of early stability due to its high density, early revascularization and osteoinduction may be sacrificed. Osteoclasts are required to first reabsorb bone, making way for the formation of cavities to the osteonal canal. Upon reaching the canal, osteoblasts are then able to start bone formation. Eventually, this results in complete resorption of graft and replacement with new bone [10]. In contrast, cancellous bone is very osteogenic due to its large surface area. Osteoblasts are able to rapidly incorporate new bone and revascularization happens relatively quickly when compared to cortical grafts. Although early mechanical strength is limited, the ability to rapidly begin producing new bone generally outweighs the risks in most patients [10].

While the advantage of being able to promote strong fusion with complete histocompatibility has firmly established its widespread usage, autografts are not without drawbacks. The quality of individual grafts can vary according to age and metabolic activity [13]. Patients undergoing extraction of autografts also have a risk of suffering from blood loss and pain at the donor site [14]. Therefore, these complications encouraged the development of autograft substitutes.

#### 3.1.1. Iliac Crest Bone Grafts (ICBGs)

**Figure 1:**
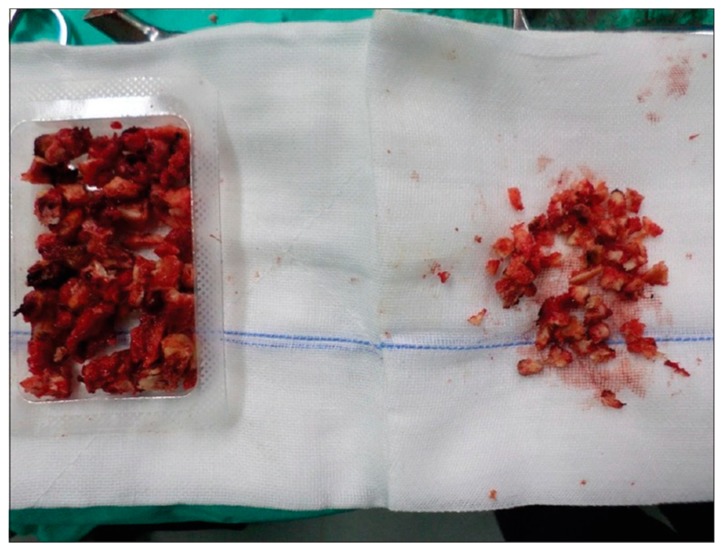
Autogenous bone graft prepared from a local bone source during neural decompression. Reproduced under the Creative Commons Attribution (CC BY) license from Boktor J, Ninan T, Pockett R, Collins I, Sultan A, Koptan W. Lumbar fusion for lytic spondylolisthesis: Is an interbody cage necessary? *Journal of Craniovertebral Junction & Spine.* 9(2): 101–106, 2018 [18].

As previously stated, the iliac crest is the most common site for autograph harvest [15]. However, there are many reported complications of harvesting from the iliac crest including the development of infections, seromas, hematomas, and fractures to the iliac spine with reported complication rates of 1.40%, 0.64%, 1.49%, and 0.16%, respectively, with some requiring further operative management [12,16]. Graft site hernias are another rare complication [17]. Iatrogenic nerve injuries were not uncommon either, with reported complication rates of 0.31% to the cluneal nerve and 0.68% to the lateral femoral cutaneous nerve. Sensory disturbance occurred in 4.81% of patients [12]. Further, Dimitriou et al. [12] found a total complication rate of 19.37% for extracting iliac crest bone grafts (ICBGs). Because of these complication rates, ICBGs have generally fallen out of favor for remote graft harvests but may still be utilized when crest exposure is part of a lumbar fusion wound. Additionally, new methods of harvesting bone have been developed for local bone harvesting such as the creation of the Reamer/Irrigator/Aspirator (RIA) (Synthes Inc., West Chester, PA, USA). By utilizing the RIA to harvest bone graft from the intramedullary canal located between the femur and the tibia, surgeons were able to effectively lower complication rates of extracting autografts to a mere 6% [12]. A preparation of autogenous graft from local bone is shown in Figure 1.

#### 3.1.2. Bone Marrow Aspirates (BMAs)

Due to the drawbacks and morbidities of extracting ICBGs, some surgeons have utilized bone marrow aspirates (BMAs) along with scaffolding as a replacement of ICBGs for spinal fusion. BMA is a cellular-based graft containing both osteoprogenitor and hematoprogenitor cells harvested directly from the host’s posterior iliac bone. It can be easily harvested with large bore BMA needles with minimal donor site morbidity. Due to its lack of osteoconductive ability, it is often combined with allografts [19]. A meta-analysis by Khashan et al. found that the fusion rates of BMAs incorporated with scaffolding were similar when compared to autografts without scaffolding with rates of 100% and 96.7%, respectively [20,21].

### 3.2. Allografts

Allografts involve the transplantation of bone from one individual to another. They are typically obtained from either a cadaver or a living donor (such as after a hip replacement surgery) when autografts from the patient are unobtainable. Aside from being readily available, allografts have an additional advantage of lacking the need for multiple incision sites from the patient to harvest the graft [22]. Recently, the focus on incorporating MSCs into allografts has increased the efficacy of these grafts [13]. Like autografts, allografts are classified as either cortical or cancellous [10].

Allografts often require sterilization, with the standard method involving gamma radiation. The goal of gamma radiation is to eliminate the risk of disease transmission by destroying microorganisms, which have been widely proven to be able to effectively inactivate pathogens, while ideally having the lowest possible impact on structural integrity of the tissues [23]. However, the sterilization process can damage the molecular structure of fragile biologics such as cytokines, chemokines, and growth factors which can alter the biomechanical properties of bone [24]. Gamma radiation has several advantages of other methods that include better penetration, greater certainty of sterility, and effectiveness that is independent of temperature and pressure [24].

There are some disadvantages to allografts. Because allografts are also derived from human origin, they are both osteoconductive and weakly osteoinductive. However, because of the sterilization process, allografts lack viable cells and have no osteogenic properties [25]. Other disadvantages of allografts include the limited risk of HBV or HCV infection from the donor and the potential of adverse changing of the bone matrix composition during the process of sterilization with chemicals and radiation [22,26].

Comparative studies between allografts and autografts have demonstrated similar fusion rates between the two. A large retrospective case-controlled study by Suchomel et al. that involved 113 patients who underwent posterolateral fusion found a fusion rate of 94.6% and 93.4% for autografts and allografts, respectively [27]. A recent systematic review conducted by Liao et al. investigated the comparison of allografts to autografts in patients undergoing lumbar fusion. In the reviewed articles, fusion rates were not significantly different (OR = 0.567, 95% CI = 0.15–2.17; *p* > 0.05) [28]. Further, the authors deemed the use of allografts a good alternative to autografts because of their similar fusion rates, Oswestry Disability Index (ODI) scores, and visual analogue scale (VAS) pain scores. Even though multiple other studies have also demonstrated similar fusion rates between the two types of grafts, autografts still remain the gold standard due to their relatively shorter time to complete fusion [29,30].

### 3.3. Demineralized Bone Matrices (DBMs)

Demineralized bone matrices (DBMs) are organic allografts from which the mineralized portion is acid-extracted, leaving behind the organic matrix, which is made up of collagenous and non-collagenous proteins, and growth factors. DBMs are available for surgeons to use in a variety of forms ranging from putties to pastes to injectable gels [7]. DBMs’ osteoconductive properties arise from the scaffolding provided by collagenous and non-collagenous proteins preserved throughout the initial treatment of the allograft. Growth factors such as bone morphogenetic protein (BMP), fibroblast growth factor, and transforming growth factor beta (TGF-β) confer its osteoinductive properties [7].

Due to the increasing number of DBM manufactures and resulting various preparation methods, there is large variability in BMP levels among different grafts, thus making the efficacy of DBMs difficult to ascertain in clinical studies. A randomized clinical trial by Kang et al. found rates of successful fusion at two year follow up to be 92% and 86% for autograft and DBM, respectively [31]. Another study by Kim et al. found fusion rates at two years to be 62.2% and 52% for autograft and DBM, respectively [32].

**Figure 2:**
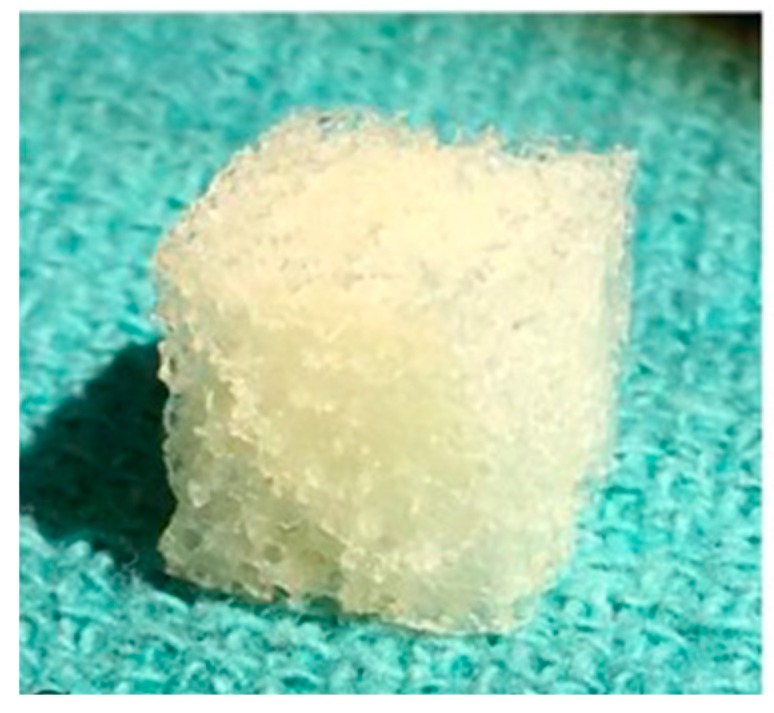
Commercial Demineralized Bone Matrix (CONFORM^®^ FLEX Demineralized Cancellous Bone, DePuy Synthes, Distributed by Musculoskeletal Transplant Foundation). Reproduced and modified under the Creative Commons Attribution (CC BY) license from Bracey D, Seyler T, Jinnah A, Lively M, Willey J, Smith T, Van Dyke M, Whitlock P. A Decellularized Porcine Xenograft-Derived Bone Scaffold for Clinical Use as a Bone Graft Substitute: A Critical Evaluation of Processing and Structure. *Journal of Functional Biomaterials.* 9(3): 45, 2018 [36].

Further clinical trials are needed to assess the risk factors of using DBMs over autografts. There is concern that DBMs carry a higher risk of graft collapse when compared to autograft due to inferior structural composition [33]. The use of contaminants in DBMs is also a concern, albeit a limited one because of current United States Food and Drug Administration (FDA) processing guidelines. One such contaminant is ethylene glycol, a known cause of acute tubular necrosis [34]. Although the use of DBMs with additional growth factors provides an avenue for future innovative research, DBMs combined with autografts currently yield the most efficacious results overall [35]. A preparation of demineralized bone matrix is displayed in Figure 2.

## 4. Bone Graft Substitutes and Supplements

### 4.1. Ceramics

**Figure 3:**
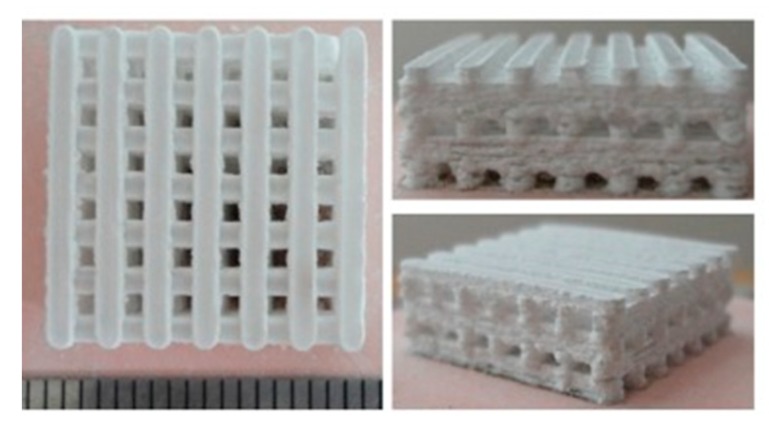
Bioactive ceramic scaffolding produced by the method of selective laser sintering. Reproduced and modified under the Creative Commons Attribution (CC BY) license from Gao C, Deng Y, Fend P, Mao Z, Li P, Yang B, Deng J, Cao Y, Shuai C, Peng S. Current Progress in Bioactive Ceramic Scaffolds for Bone Repair and Regeneration. *International Journal of Molecular Sciences.* 15(3): 4714–4732, 2014 [38].

Ceramics have been employed in orthopedics since the 1970s and can be divided into non-ceramic and ceramic hydroxyapatite. Based upon the natural occurring calcium salts and hydroxyapatite found in human bone, ceramics are synthetic grafts able to provide osteoconductive support for fusion [7,37]. Ceramic scaffolds with hydroxyapatite are the most frequently used as hydroxyapetite acts as an excellent carrier for various osteogenic cells and growth factors. With the supplementation of osteogenic cells or growth factors, this allows for both osteoinductive and osteoconductive capabilities [37]. A preparation of ceramic scaffolding is displayed in Figure 3.

Advantages of using ceramics include its long shelf life, virtually zero risk for disease/virus transfer, ease of being manufactured, and the ability to be pre-formed into a desirable shape for the patient. They can also be effective as bone graft extenders in posterolateral fusions in which they are currently most used clinically [39,40]. Disadvantages, on the other hand, include its lack of cortical stability and osteogenic properties [25]. Due to the limited supply of autografts and allografts, ceramics can be optimized with different growth factors to provide a cheaper, more easily manufacturable alternative to these types of grafts [5]. An overview of the capabilities of each bone graft and bone graft substitute are provided in Table 1.

### 4.2. Polyetheretherketone (PEEK)

**Figure 4:**
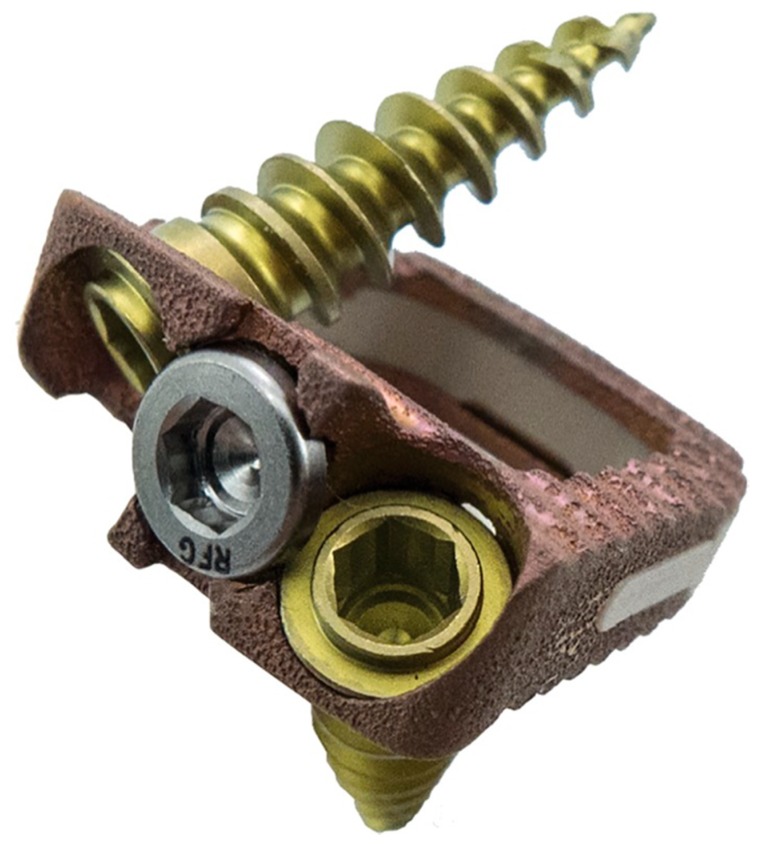
Redmond (A-Spine, Asia, Taiwan, China), composite titanium (Ti)/polyether ether ketone (PEEK) integral fixation spacer featuring ridged titanium alloy endplates in combination with a PEEK body. Reproduced under the Creative Commons Attribution (CC BY) license from Phan K, Pelletier MH, Rao PJ, Choy WJ, Walsh WR, Mobbs RJ. Integral Fixation Titanium/Polyetheretherketone Cages for Cervical Arthrodesis: Evolution of Cage Design and Early Radiological Outcomes and Fusion Rates. *Orthopaedic Surgery*. 11(1): 52–59, 2019 [43].

The biocompatible polymer, polyether ether ketone (PEEK), was first introduced in the 1990s by AcroMed as a spinal cage for the facilitation of spinal fusion [41]. It was found to be a comparable alternative to autographs for spinal fusions. PEEK Cages are radiolucent and have a low elastic modulus, making them attractive alternative. However, they still come with the potential for complications such as pseudarthrosis, subsidence, and migration of the cages [42]. An example of a PEEK interbody cage is displayed in Figure 4**.**

When discussing outcomes for PEEK cages in spinal fusions, a systematic review found minimal evidence for better clinical and radiologic outcomes compared to bone grafts in the cervical spine. There was no difference found between PEEK, titanium, and carbon fiber cages [42]. A meta-analysis including six studies comparing anterior cervical discectomy and transforaminal interbody fusion found no difference between fusion rates of PEEK cages and titanium cages, but did note that there was a higher subsidence rate with the titanium cages [44]. PEEK cages can be combined with ceramics for additional osteoconductive effects and have been shown to be a suitable substitute for autograft in anterior cervical discectomy and fusion [45]. Another study examined fusion rates of allogenic cancellous bone vs. cancellous iliac crest autograft in combination with PEEK cages for instrumented monosegmental lumbar spondylodesis and found no significant difference between fusion rates: 80% and 85%, respectively [29]. Although PEEK cages have been found to have similar and adequate outcomes compared to the alternatives, future randomized studies are still needed to further establish equivalency.

### 4.3. Bone Morphogenetic Proteins

Bone morphogenic proteins (BMPs) were first isolated in 1965 by Robert Urist, and have since been extensively studied for their clinical application in spinal fusion [7]. The term BMP refers to over 20 known cytokines and growth factors of the TGF-β family with osteogenic capabilities. Of this family, BMP-2, BMP-4, and BMP-7 (osteogenic protein-1) are the most studied [46,47]. BMPs also widely used in other areas in medicine including dental treatment, cancer, and in open tibial fractures [48].

BMP signaling utilizes both BMP type I and type II receptors in order to initiate downstream mediators, most notable of which is the SMAD pathway. The SMAD pathway involves the use of cytoplasmic transcription regulators of chromatin remodeling machinery and the expression of tissue-specific transcription factors. Thus, the BMP family of growth factors is involved with osteoinduction and the resulting endochondral ossification [46]. Furthermore, the rise of genetic cloning capabilities has made it possible to produce large quantities of BMPs in order to promote more effective bony fusion in patients [7].

BMPs can be used either alone as bone graft substitutes with a synthetic collagen carrier or in addition to other autograft or allograft materials. The ability of BMPs to enhance bony fusion has been confirmed by comparative trials. A meta-analysis by Parajón et al. comprising of 40 studies found that fusion rates with the use of recombinant human bone morphogenetic protein (rhBMP) were slightly superior compared to fusions without the use of rhBMP (96.6% and 92.5%, respectively) [6]. They found the highest rate of fusion in cases where rhBMP was used in combination with local bone autograft (99.1%). Although autografts still remain the gold standard, rhBMP serves as a potential addition to these grafts to increase fusion rates. One study found that BMP can be further augmented to promote spinal fusion when delivered with either basic fibroblast growth factor, FK506, elcatonin, and hyperbaric oxygenation [49].

Interestingly, recent evidence suggests that BMP levels at supraphysiological levels show no beneficial effect in spinal fusion patients [50]. Chan et al. have also found high rates of BMP inhibitors that are expressed by and mesenchymal stem cells such as chordin, gremlin1, gremlin 2, follistatin, and noggin [51]. They suggest that treatment modalities can be developed to target these antagonists and produce a stronger spinal fusion. One study found increased mitochondrial activity of mesenchymal stem cells when incubated with intervertebral disc cells [52]. BMP antagonists were also found to be upregulated in nucleus pulposus cells, annulus fibrosa cells, and cartilaginous endplate cells [53].

Over the last decade, some potentially severe complications have been reported with the use of rhBMP, including dysphagia and airway complications necessitating respiratory support [54]. In a study involving 38 patients treated with a multi-level ACDF using rhBMP, Khajavi and Shen reported two cases that were readmitted and given steroids due to worsening dysphagia and/or excessive prevertebral swelling with concern for major airway compromise. When used in transforaminal approaches, it is known that rhBMP can lead to bony overgrowth, resulting in nerve root compression [55,56]. These adverse effects have led to an FDA-issued warning for the use of rhBMPs in fusion procedures of the cervical spine [57]. Another notable complication of BMP is their implication in oncogenesis. They have been found to potentiate malignancies of several types of tumors while suppressing others [58]. Kokorina et al. [59] found rhBMP-2 to have an adverse biological effect on invasiveness of human oral squamous cell carcinoma cell lines in vitro. High doses of rhBMP-2 have also been loosely correlated with increased rates of deep infections (2.4%), arrhythmias (2.4%), cancer (3.4%), and pseudarthrosis (5%) in certain studies [48,60]. In an attempt to better alleviate risk of these complications, some studies with animal models suggest using parathyroid hormone (1–34) in addition to BMP to lessen the amount of BMP dosage required [61,62,63]. Finally, the cost of BMP is high according to one economic evaluation, and it may not be cost-effective for use in the majority of patients [64].

## 5. Autologous Growth Factors (AGFs)

Platelet degranulation leads to the release of growth factors that contribute to both bone and wound healing. These autologous growth factors (AGFs) contain mitogenic properties for inducing proliferation of osteoblasts, fibroblasts, and mesenchymal stem cells [65]. Two of the most researched growth factors include platelet-derived growth factor (PDGF) and TGF-β. PDGF is thought to directly increase the replication and synthesis of matrix proteins, playing an important role in the remodeling and construction of new bone [65]. Similarly, TGF-β regulates extracellular bone matrix synthesis and serves a crucial role of stimulating angiogenesis [11]. These growth factors are extracted and prepared via the ultra-concentration of platelets, and theoretically can be used in combination with either autograft, allografts, or ceramics in order to increase rates of successful fusion [66]. Further, platelet-rich plasma is utilized in a variety of other orthopedic procedures as well including rotator cuff tears, tendinopathies, osteoarthritis, and articular cartilage injuries [67].

While there has been much basic science research supporting the role of AGFs in bone formation and remodeling, clinical data thus far has not endorsed any ability of AGFs to increase spinal fusion rates compared to traditional autograft [5,68]. While Jenis et al. found similar fusion rates comparing allograft with AGFs compared to autografts alone, these authors promote the use of AGFs with allografts in order to eliminate the need for iliac graft harvesting [69]. It is also important to consider the increased financial costs of blood draws and laboratory processes that are required for preparing AGFs [70].

## 6. Mesenchymal Stem Cells (MSCs)

Adult mesenchymal stem cells (MSCs) are currently widely used in the repair and regeneration of damaged tissues [71]. MSCs are capable of differentiating into osteoblasts and chondrocytes, thereby making them a viable option for utilization in spinal fusion [72]. These stem cells are most commonly harvested from the iliac crest and deposited within grafts to enhance new bone formation. This is performed by fine needle puncture. A battery powered drill is then used to drill the trochar and needle into the cortical bone. The bone marrow aspirate (BMA) is then filtered and centrifuged, suspended in platelet poor plasma, and then, hemoanalysis and complete blood count with differential is performed to analyze the product before use [73]. By combining harvested stem cells with ceramics and allografts, one can create a graft with the three properties formerly only possessed by autografts: osteogenesis, osteoinduction, and osteoconduction [74].

The utility of MSCs has been demonstrated in clinical research and has been found to be as efficacious as the use of rhBMP in combination with grafting. Patients undergoing MIS-TLIF with MSCs had similar rates of fusion and revision surgery compared to patients undergoing fusions with rhBMP-2 and allograft [75]. With regards to side effects, the use of MSCs has been found to result in chronic harvesting site pain [15,16]. One way to minimize this complication would be to harvest local grafts from the surgical site itself. Local harvesting has been shown to contain more fibroblastic colony forming units than iliac crest. This property could help extract MSCs more efficiently in addition to minimizing donor site pain.

## 7. Synthetic Peptides

P-15 is a synthetic peptide consisting of a 15-amino acid sequence found in the residues of the alpha-1 chain of type I collagen [76]. Because of its biomimetic capabilities, P-15 is able to enhance bone mineralization when used in combination with anorganic bone mineral (ABM) [4,77]. ABM is a collection of calcium phosphate granules that provide the scaffolding and source of calcium for bone formation and thus has strong osteoconductive properties. A novel bone graft substitute called i-FACTOR^TM^ (Cerapedic Inc, Westminster, CO) is made up of a combination of these two materials (ABM and P-15) suspended in a hydrogel carrier [76].

Focused on i-FACTOR outcomes, Mobbs et al. demonstrated radiographic evidence of bony induction and early incorporation of bone grafts [78]. Fusion rates were 97.5%, 81%, and 100% for single-level fusions, two-level fusions, and three-level fusions, respectively, at a two-year follow up. Additionally, there was a statistically significant improvement in the postoperative disability index [78].

Because i-FACTOR has only recently been introduced to the market, there are only few third-party studies comparing the fusion rates of i-FACTOR to the traditionally used autograft. Recent studies have shown that fusion rates with PEEK interbody cages filled with i-FACTOR versus PEEK interbody cages filled with autogenous bone are comparable. Fusion rates of i-FACTOR compared to autograft in anterior cervical discectomy and fusion were slightly higher, and intra-cage bridging with i-FACTOR occurred earlier than autograft in posterior lumbar fusions [79,80,81]. Additional research is needed to justify the use of this novel product over autograft for spinal fusion.

## 8. Gene Therapy

One of the most innovative research initiatives in spinal fusion involves the targeting and expression of genes encoding osteoinductive and osteogenic factors. Targeting these genes via viral transduction could theoretically allow cells to release more growth factors into the extracellular environment for the purpose of obtaining maximal bone growth [80]. This method has been largely successful in animal models, where both BMP-2 and BMP-9 have been injected with subsequent bone formation [46,80,82].

An issue with gene therapy, however, is that it is difficult to assess for successful gene transduction in vivo, and hence difficult to assess its clinical efficacy in patients [80,83]. As a solution to this issue, cells are beginning to be transduced ex vivo [80,84]. Autogenous cells are extracted from the donor and are cultured on laboratory media. They are then transduced with a viral vector, and the amount of protein expression is measured. After sufficient growth factor has been expressed and quantified, the autografts are then implanted back into the donor. An advantage of this ex vivo approach is that transduced cells can easily be isolated and expanded for a more efficient production of growth factors. This ex vivo approach has shown success in rat models [85].

Of the potential viral vectors possessing transduction capabilities, adenoviruses are currently the most utilized in trials of bone healing due to their high transfection capacity [82,86]. Disadvantages of the adenoviral vector include limited protein production, as the vector is unable to integrate into the host’s genome. Further, adenoviruses elicit a large immune response [87]. Though this method of viral transduction for gene therapy appears promising for the future, it is important that these viral vectors are studied long-term for safety and efficacy before their introduction into clinical practice. An overview of all spinal fusion supplements is provided in Table 2.

## 9. Conclusions

Iliac crest autografts remain the gold standard for interbody grafts in spinal fusion. However, as the field of biologics and grafts becomes increasingly innovative, the number of options to choose from continues to rise. Surgeons can avoid the donor site complications that comes with autografts by instead using one of the many allografts, DBMs, or synthetic ceramic products currently on the market. Furthermore, there is strong evidence that proteins can be used in combination with grafts to improve rates of successful fusion in patients. Last but not least, genetic therapy used in the stimulation of growth factor synthesis has demonstrated success in preliminary animal models. Further scientific effort should be encouraged for the development of more efficient grafting techniques and biologics to achieve the best rates of fusion for patients.

## Figures and Tables

**Table 1:** Overview of the Osteoconductive, Osteogenic and Osteoinductive Properties of Graft Materials and Bone Graft Substitutes. Capabilities of each graft and graft substitute along with advantages and disadvantages of the grafts as provided in the current literature. MSC = Mesenchymal Stem Cell; HBV = Hepatitis B Virus; HCV = Hepatitis C Virus.

Graft Material	Osteoconductive Capability	Osteogenic Capability	Osteoinductive Capability	Advantages	Disadvantages
Autogenous Bone Grafts	Yes	Yes	Yes	Complete histocompatibility, no opportunity for infection from graft donor, and promotes strong fusion as being the only graft having all three pillars of spinal fusion	Quality of graft is dependent on patient age and metabolic activity [13], and there is a risk of blood loss and local pain at the extraction site [14]
Bone Marrow Aspirates	No	Yes	Yes	Minimal donor site morbidity	Must be incorporated with scaffolding or allografts [18]
Allografts	Yes	No	Yes	Lacks donor site morbidity and can be combined with MSCs [13]	Requires sterilization, process of sterilization alters the biomechanical properties of bones [22], and there is a possible risk of infection with HBV or HCV [21]
Demineralized Bone Matrices	Yes	No	Yes		Lower fusion rates than autogenous grafts [30,31], higher rates of spinal collapse [32], and suffers from possible contamination during its production process [33]
Ceramics	Yes	No	No	Can be supplemented with osteogenic cells and growth factors [35], has a long shelf life, has zero risk for disease transfer, and is easily and cheaply manufactured [36,37]	Lacks cortical stability and osteogenic properties [24]

**Table 2:** Overview of Bone Graft Supplements. Mechanism of each supplement is provided along with advantages and disadvantages as provided in the current literature. TGF-β = transforming growth factor-beta; BMP = bone morphogenetic protein; MSC = mesenchymal stem cell.

Supplements	Mechanism	Advantages	Disadvantages
Bone Morphogenetic Proteins	TGF-β family cytokines that initiate SMAD pathway activating transcription factors for growth [7]	Enhances osteogenesis and oseoinduction [42], and genetic cloning capabilities make it possible to produce in large quantities [7]	At supraphysiological levels they are antagonized by BMP inhibitors [47], can cause dysphagia and airway complications [50], it is potentially oncogenic [54], and has high costs [48]
Autologous Growth Factors	Growth factors that are released from platelet degranulation that activates the proliferation of osteoblasts, fibroblasts and MSCs [61]	Can be used with autografts, allografts or ceramics to increase rates of successful fusion [62]	There is no clinical data as of this time that provides definitive evidence of an increase in the rate of spinal fusion [5]
Mesenchymal Stem Cells	Differentiate into osteoblasts and chondrocytes to promote spinal fusion [68]	Can create a graft with all the properties of osteogenesis, osteoinduction and osteoconduction [70]	Potentially leads to chronic harvesting site pain [15]
Synthetic Peptides	Amino acid sequences found in alpha-1 chains of type I collagen that enhances bone mineralization [72,73]	Fusion rates of i-FACTOR compared to autograft were slightly higher in some studies [75,76,77]	To date, there is still minimal third-party studies measuring rates of fusion
Gene Therapy	Targeting the expression of genes that encode osteoinductive and osteogenic factors [76]		Difficult to assess successful gene transduction in vivo, and, thus, its performance is difficult to measure in clinical trials [76]
